# HirBin: high-resolution identification of differentially abundant functions in metagenomes

**DOI:** 10.1186/s12864-017-3686-6

**Published:** 2017-04-21

**Authors:** Tobias Österlund, Viktor Jonsson, Erik Kristiansson

**Affiliations:** Department of Mathematical Sciences, Chalmers University of Technology and University of Gothenburg, SE-41296 Gothenburg, Sweden

**Keywords:** Metagenomics, Next-generation sequencing, Functional annotation, Binning, TIGRFAM, Differential abundance, Statistical analysis

## Abstract

**Background:**

Gene-centric analysis of metagenomics data provides information about the biochemical functions present in a microbiome under a certain condition. The ability to identify significant differences in functions between metagenomes is dependent on accurate classification and quantification of the sequence reads (binning). However, biological effects acting on specific functions may be overlooked if the classes are too general.

**Methods:**

Here we introduce High-Resolution Binning (HirBin), a new method for gene-centric analysis of metagenomes. HirBin combines supervised annotation with unsupervised clustering to bin sequence reads at a higher resolution. The supervised annotation is performed by matching sequence fragments to genes using well-established protein domains, such as TIGRFAM, PFAM or COGs, followed by unsupervised clustering where each functional domain is further divided into sub-bins based on sequence similarity. Finally, differential abundance of the sub-bins is statistically assessed.

**Results:**

﻿We show that HirBin is able to identify biological effects that are only present at more specific functional levels. Furthermore we show that changes affecting more specific functional levels are often diluted at the more general level and therefore overlooked when analyzed using standard binning approaches.

**Conclusions:**

HirBin improves the resolution of the gene-centric analysis of metagenomes and facilitates the biological interpretation of the results. HirBin is implemented as a Python package and is freely available for download at http://bioinformatics.math.chalmers.se/hirbin.

**Electronic supplementary material:**

The online version of this article (doi:10.1186/s12864-017-3686-6) contains supplementary material, which is available to authorized users.

## Background

Metagenomics is the study of microbial communities by high-throughput sequencing of the DNA present in a sample. The use of metagenomics has accelerated during the last couple of years following the technological advances in next-generation sequencing, resulting in large amounts of data being generated [1]. Metagenomics has, for example, enabled exploration of the structure and diversity of microbial communities in their natural habitat, both for the human microbiota [2, 3], and in the environment [4]. Due to the nature of metagenomics the data often shows a high diversity, low coverage and a high rate of sequencing errors, while the generated sequence reads are short. This makes the data processing and analysis important in order to draw correct conclusions from the data. In gene-centric analysis metagenomic data is quantified based on the gene content, which provides information about the abundance of biochemical properties and pathways [5, 6]. In this process, sequence reads are first aligned to annotated reference sequences and then sorted (‘binned’) based on the function of their matching genes [7]. Each bin is then quantified based on the total number of matching reads. Identification of differentially abundant genes, pathways and functions can, consequently, be performed by statistical comparison of the abundance of the bins between metagenomes sampled from different environments or conditions [8].

Functional binning of metagenomes is today a supervised process where a reference sequence or the sequence reads are annotated using sequence homology, often using profile Hidden Markov Models (HMM) or position-specific weight matrices. Several computational methods have been developed for this purpose, including MG-RAST [9], Megan4 [10], COGNIZER [11], Medusa [12], Tentacle [13], CloVR [14] and MOCat2 [15]. These methods differ in their approach to read mapping and reference databases used for annotation. The gene profiles used for annotation are typically from databases such as PFAM [16], TIGRFAM [17], FOAM [18] or COG [19]. Annotation using PFAM and TIGRFAM are based on defined gene or protein families, the FOAM database is built using KEGG orthologies (KOs) [20] while COGs are based on clusters of orthologous groups. Sequenced-based functional annotation has earlier been reviewed [21]. The gene profiles used for annotation using these different databases are typically broad and designed to be able to identify as many protein variants in as many species as possible [16, 22]. As a consequence, many domain models provides a general functional classification, but lacks the ability to discriminate between more specific functional differences. As an example, there are around 10 million genes described in the human gut microbiome [23], while the total number of bacterial PFAM protein families is only 9,495 (PFAM release 29.0), and the total number of TIGRFAMs is 4284 (TIGRFAM v.13.0). It is based on these numbers clear that binning based on PFAM or TIGRFAM protein families will group a large number of different genes into a single bin. When comparing gene abundances between metagenomes from different conditions it is therefore a risk that specific functions with important differences between conditions are mixed with other functions that are of less interest. This will affect the statistical power negatively by decreasing the signal to noise ratio and make the true differences harder to detect. The exact impact of this ‘dilution’ effect has however not been investigated, and the consequences are therefore not known. In order to address this knowledge gap we asked two questions: When comparing metagenomes from different conditions, given annotation using commonly used functional domains (e.g., PFAM), 1) which effects (differential abundance between metagenomes) can be found at a more specific functional level, and 2) are those effects overlooked when comparing the metagenomes at the general functional level?

To answer these questions, and to be able to detect changes at a more specific functional level, we have developed a new method for gene-centric analysis of metagenomes, called HirBin. The method uses a data-driven binning approach which extends existing methods (using e.g., annotation with TIGRFAMS, PFAMS or COGs) by adding a second unsupervised binning step to find more specific sub-bins. First, HirBin performs binning using supervised annotation of known functional domains, which is then followed by unsupervised clustering to identify the sub-bins that defines functions at a more specific level. Statistical analysis is then performed on the sub-binned level which enables identification of functional differences with a higher resolution. By analyzing data from the human gut we show that HirBin is indeed able to identify effects that are present on more specific functional levels. Furthermore, we also show that effects acting on more specific levels are often diluted on the more general levels, and therefore overlooked when using standard approaches for gene-centric analysis of metagenomes, e.g., annotation using gene profiles or orthologous groups. HirBin is freely available as a python package and designed to run in parallel in a Linux environment.

## Results

### A novel method for refined functional annotation and statistical analysis

**Fig. 1:**
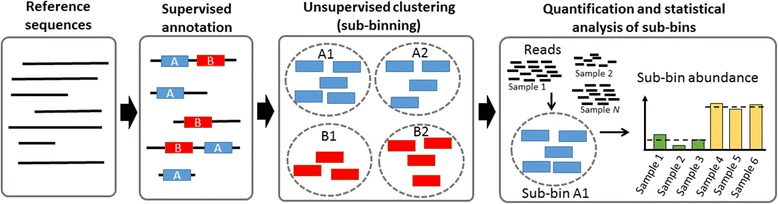
An overview of the HirBin workflow. HirBin uses reference sequences as input to the annotation step. The reference sequences are annotated in terms of known functional domains such as TIGRFAM, PFAM or COG (supervised annotation). Each annotated domain is then clustered based on sequence similarity (unsupervised clustering) forming sub-bins. The sub-bin abundance is then calculated by mapping the reads in each sample to the sub-bin sequences and finally differentially abundant sub-bins between conditions are identified by statistical analysis

We have developed a new method for improved functional binning and identification of differentially abundant functions in metagenomes. The method, called HirBin (High-Resolution Binning), extends previous binning methods by a second sub-binning step, combining an initial supervised functional binning step with an unsupervised sub-binning step in order to improve the resolution in the binning process for large-scale metagenomes. As a consequence HirBin enables identification of differentially abundant genes at a higher functional resolution. In this context, we use ‘bin’ to denote a set of sequence reads that are associated with genes predicted to have similar biological function and/or biochemical properties. Analogously, we define a ‘sub-bin’ as any subset of a bin for which the reads are predicted to be associated with a more specific function. The sub-bins are created from the bins by unsupervised clustering of the sequences in each bin based on sequence similarity. The HirBin workflow is presented in Fig. 1 and consists of four main analysis steps. The input to the analysis are reference sequences in FASTA format (e.g., assembled contigs or a collection of reference genes or genomes) and sequence reads in FASTQ format. The output is a list of sub-bins that have a significant change in their abundance between the studied conditions. In the supervised annotation step (functional binning step) HirBin annotates the reference sequences using protein domain profiles or orthologous groups. HirBin supports multiple profile databases, including TIGRFAM [17], PFAM [16] and COGs [19]. It is also possible to provide HirBin with user-defined annotations of the reference sequences. In the unsupervised annotation step (sub-binning step) the amino acid sequences of the annotated domains are clustered based on sequence similarity, using a provided sequence similarity cutoff, which generates the sub-bins. The sub-bins are then quantified by mapping the reads to the annotated reference sequences at the nucleotide level and counting the number of matches for each sub-bin. Finally, HirBin performs a statistical analysis using an overdispersed Poisson count model to identify differentially abundant sub-bins between different conditions. See Methods for complete details regarding the implementation of HirBin.

### HirBin can identify metagenomic changes with a more detailed resolution

**Fig. 2:**
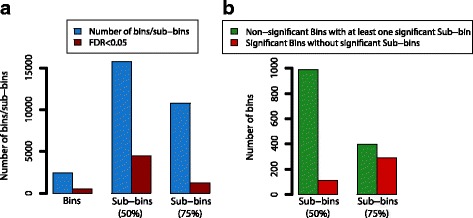
Number of bins and sub-bins. **a** The *blue* bars show the total number of bins (without clustering) for the type 2 diabetics dataset, and sub-bins when clustering using 50 and 75% sequence identity cutoffs. The red bars show the number of significant bins or sub-bins (FDR < 0.05) when comparing type 2 diabetic patients to control. **b** The number of bins (TIGRFAMs) that have at least one significant sub-bin (FDR < 0.05) at the sub-bin level, but are not significant at the bin level (*green* bars) and the number of bins that are significant (FDR < 0.05) both at the bin level and the sub-bin level (*red* bars)

In order to demonstrate the ability of HirBin to identify differentially abundant functions, we analyzed data from a human gut metagenomics dataset with our method, comparing the metagenome of 15 patients with type 2 diabetes (t2d) with 15 controls [24]. The aim was to identify functions changing in abundance between the two groups. The assembled metagenomes from the selected individuals were annotated using TIGRFAM functional domains to define bins, which were then further divided into sub-bins. We performed functional annotation with HirBin using two different sequence similarity cutoffs (Fig. 2). The sub-binning at 75% amino acid sequence similarity represents a stricter functional annotation while the clustering at 50% is less strict, grouping a higher number of sequences together in each sub-bin. The analysis using HirBin (at 50 and 75% sequence similarity) was compared to the analysis using TIGRFAM annotations without sub-binning (bins). Furthermore, only bins and sub-bins detected in at least 75% of the samples and thus representative for the full data set were considered in the analysis (see Methods). Fig. 2a shows the total number of representative bins and sub-bins. The fold changes and false discovery rates of all bins and sub-bins at the different sequence similarity cut-offs are presented in Additional file 1. The number of detected representative sub-bins were 15,740 at the 50% and 10,798 at the 75% sub-binning level, compared to the number of detected bins (TIGRFAMs) that were 2,465. The fact that the number of sub-bins is lower at 75% identity than at 50% identity is a result caused by the lack of representative sub-bins at stricter clustering cut-offs and reflects the high diversity often seen between gut metagenome from different individuals [25, 26]. Statistical analysis comparing individuals with and without t2d showed that 457 of the 2,465 (18.5%) original bins were differentially abundant (FDR < 0.05, left-most bar in Fig. 2a). In comparison, at the less strict clustering level (50% sequence similarity.), 4,436 (28.2%) of the sub-bins were significantly differentially abundant between the two groups (FDR < 0.05). The proportion of significant sub-bins decreased at the stricter sequence identity cutoff (75%), which resulted in 10,798 sub-bins, of which 1,248 (11.6%) were significant.

A large proportion of the significant sub-bins corresponded to bins that were not significant at the bin (TIGRFAM) level. At a 50% sequence similarity, 987 (48.5%) non-significant bins had at least one sub-bin with a significant differential abundance among the t2d patients compared to control (Fig. 2b). The analysis at the sub-bin level also showed that 112 of the 502 significant bins did not have any significant sub-bin. Thus, at a 50% sequence identity cutoff, HirBin identified 987 additional bins where the significant effect only could be identified at a more specific level while the effect was lost for 112 bins. The 112 bins that were not detected at the sub-bin level had a lower average abundance compared to other bins, suggesting that their significance was lost due to too low power (Mann-Whitney *U*-test *p*-value < 2.2*10^−16^, Additional file 2). When the identity cut-off was increased to 75%, the difference between the non-significant bins with a significant sub-bin and the significant bins without a significant sub-bin decreased (396 bins that gained significance and 292 bins that lost significance). This is likely due to the decreasing number of representative sub-bins represented in at least 75% of the individuals at the 75% identity cut-off.

To investigate if significant sub-bins provided additional biological knowledge, we performed a gene set enrichment analysis (GSEA) using Gene Ontology (GO) terms [27, 28] (see Methods). At the bin level, in total 21 GO terms were found to be significantly associated with genes that were differentially abundant between t2d and controls (using a strict *p* < 0.001 cut-off, Additional file 3). However, when the gene set enrichment was performed using the sub-bins instead, we observed both a considerably higher number of significant GO terms and, in general, more significant *p*-values (e.g., 56 significant terms when sub-binning using 50% sequence similarity cutoff). Interestingly, we identified many GO terms that did not show any tendency to be overrepresented at the bin level, but, were highly significant at sub-bin level. One example is the GO term associated with hydrolase activity (GO:0016787) which were not reported as significant at the bin level (*p*-value 0.621) with 6 associated bins showing an up-response (logarithmic fold change (logFC) > 0) and 5 bins showing a down-response (logFC < 0). When using sub-binning instead, this GO-term was reported as highly significant (*p*-value < 0.001) among the GO-terms showing an up-response. Here, the number of sub-bins associated with this GO-term at the 50% sequence similarity level was 22, of which 17 go up and 5 go down when comparing t2d to healthy individuals. Furthermore, there were also a large number of GO-terms that increased their significance when using HirBin, for example the GO-terms associated with transmembrane transporter activity and response to oxidative stress, which have previously been shown to be associated with t2d [24]. These two GO-terms were shown to be more significant at the sub-bin level (GSEA *p*-value < 0.001 at 50% sequence similarity) than at the bin level (*p* = 0.020 and *p* = 0.024 respectively; Additional file 3). Thus, the GSEA analysis suggests that the analysis of sub-bins provides additional biological insight into the differences of the microbiome of between healthy individuals and individuals with t2d.

### Effects seen at the sub-bin level are often diluted at the bin level

**Fig. 3:**
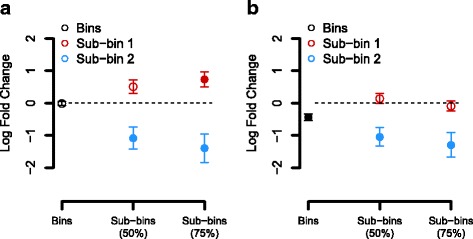
Examples of bins and sub-bins. The figure shows examples of two TIGRFAMS and selected sub-bins. The x-axis shows the percent identity cutoff used in the sub-binning step (or no sub-binning for Bins), and the y-axis shows the log fold change when comparing diabetics to control. A filled circle with error bars indicates that the sub-bin is significant (FDR < 0.05; error bars show standard error of the mean logFC). An open circle represents a non-significant sub-bin. **a** HirBin sub-binning profile for TIGR03537 (DapC: succinyldiaminopimelate transaminase). **b** HirBin sub-binning profile for TIGR01243 (CDC48: AAA family ATPase, CDC48 subfamily). For a complete picture of all sub-bins, see Additional file 4

To further investigate the impact of sub-binning when using HirBin, we analyzed two bins in more detail by studying the sub-binning profile for these bins at different sequence identity cutoffs. Figure 3a shows the bin TIGR03537 (DapC: Succinyldiaminopimelate transaminase) together with two of its sub-bins at the 50 and 75% sequence identity cut-offs (see Additional file 4 for all sub-bins of this bin). At the bin level, no significant effects were found between the two groups (FDR = 0.900) and the estimated log fold change (the average difference in abundance between t2d and control) was close to zero (−0.013, SE = 0.066). However, when the bins were analyzed at a higher resolution, the two sub-bins were found to be significant where one had positive log fold change and one negative log fold change when comparing t2d to control. At a 75% sequence similarity cut-off, sub-bin 1 had an estimated log fold change of +0.734 (SE = 0.237) with an FDR of 0.039 while sub-bin 2 had a fold-change of −1.397 (SE = 0.422) and with an FDR of 0.031. The reason why we don’t see any effect at the bin level is that the effect is diluted, i.e., sub-bins with positive fold changes and sub-bins with negative fold changes are merged and the result is an average fold change close to zero. Figure 3b shows the bin TIGR01243 (CDC48 ATPase). This is an example where the bin is reported as significant (FDR = 0.021, logFC = −0.437, SE = 0.102) and one of the sub-bins are also significant, but with even more negative log fold change. At the 75% sequence similarity clustering level the log fold change for sub-bin 2 is −1.297 (SE = 0.379, FDR = 0.029) corresponding to a 3.7-fold decrease in the t2d samples. The fact that the sub-bin level shows a more negative log fold-change than the bin level indicates that the effect is diluted at the bin level, but still detectable.

### Analysis of sub-bins improves the statistical power

**Fig. 4:**
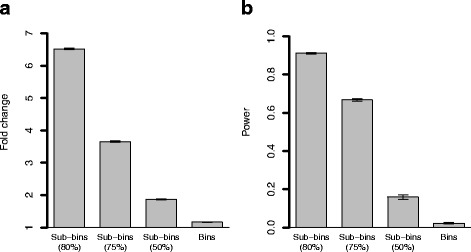
Analysis of statistical power. **a** Average fold change of regulated sub-bins at the different sub-bin levels and at the bin level. The effect is added to the 0.8 sub-bin level. **b** Power of detecting a change at the different sub-bin levels, and at the bin level. The effect is added to the 0.8 sub-bin level. The error bars show the standard error estimated from the bootstrap iterations

**Fig. 5:**
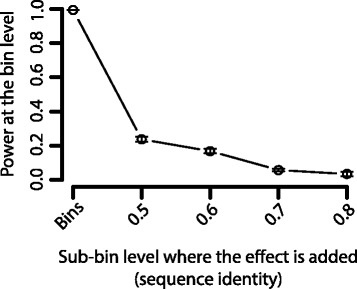
Statistical power at the bin (TIGRFAM) level (A) Power of detecting a change at the bin level (TIGRFAM level) when the effect is added at the 80, 70, 60 and 50% sequence identity sub-bin levels and the bin level. The error bars show the standard error estimated from the bootstrap iterations

In order to investigate the statistical power of HirBin in comparison to traditional binning, we performed a bootstrap analysis of a comprehensive dataset from the human gut [25] (the number of sub-bins are described in Additional file 5). An effect was added to 10% of the sub-bins at the 80% sequence similarity level (see Methods). Fig. 4 shows the calculated fold change of the bins and sub-bins with effect and the power of detecting the change with and without using sub-binning with HirBin. The results showed that the power rapidly decreases at less specific levels (Fig. 4a). Already when clustering using a 75% sequence similarity cut-off, the fold-change of the added effect had, in average, decreased by 43.9% (Fig. 4b) and, as a result, the power decreased from 91.1% at the correct level (sequence identify cut-off of 80%) to 66.8%. At 50%, the fold-change had decreased further (71.4%) and the power was as low as 15.9%. At the bin level, the effect was almost completely gone with a fold-change reduction of 82.1% and a statistical power close to 0 (2.2%). The lack of power of performing the statistical analysis at the bin level was further evaluated by repeating the above analysis but with effects added at 50, 60 and 70% respectively. The results, which are shown in Fig. 5, demonstrates that the statistical power drops rapidly. If the effect is added at the most general level for sub-binning (50%), the power dropped substantially, from 99.3 to 23.7%. This effect is caused by a strong dilution of the effects by the merging of sub-bins into bins, resulting in an average fold-change decrease of 80.4% (Additional file 6). The power is further reduced if the effect is added at more specific levels. Thus, our analysis shows that statistical inference at the bin level will mainly identify broad changes but overlook important effects introduced at more specific functional levels.

## Discussion

In this paper we present HirBin, a new method for functional annotation and identification of differentially abundant functions in metagenomes. HirBin uses a data-centric approach to improve gene quantification where bins are identified by supervised annotation using known functional domains, and then divided into sub-bins using unsupervised clustering over all samples. Due to this two-step binning procedure HirBin has the ability to identify changes between metagenomes that would be overlooked by traditional methods. In a case study, HirBin was used to compare metagenomes from individuals with type 2 diabetes to healthy controls [24]. The analysis showed that the number of sub-bins detected by HirBin was up to a five-fold larger than the more general bins (TIGRFAMs). Furthermore, a large proportion of the sub-bins were differentially abundant and many of these effects could not be identified at the bin level. A substantial number of bins that were found to be non-significant had at least one sub-bin that was significantly differentially abundant. This is a result of the broad functional classification of the bins where gene counts from multiple sub-bins are pooled. Our results suggest that effects are diluted at the bin level, due to the large number of sequences belonging to the bin that are not changing in abundance, or changing in the opposite direction, which makes the effect hard to identify in the statistical analysis. This was also underlined by the bins studied in detail (Fig. 3), which showed that significant changes present at more specific sub-bins can be masked at the bin level due to dilution of the significant effects. Furthermore, integrative analysis using GSEA showed, compared to the analysis using traditional bins, a higher number of significantly over-represented Gene Ontology terms when using sub-bins calculated with HirBin. This suggest that the increased sensitivity to detect changes in more specific functions enabled by HirBin can increase the data interpretability and provide more biologically relevant results.

The effects of dilution and the ability to detect differential abundance was further examined and quantified using resampling of an additional independent metagenomic dataset, where effects were systematically added using down-sampling of reads [8]. When effects were added to more specific sub-bins (80% sequence similarity), the estimated fold-change at the bin level was close to zero, and, as a consequence, the statistical power (the ability of the method to detect the change) was substantially reduced (97.6% decrease). When the effect was added to less specific sub-bins (50% sequence similarity) the power of detecting the change at the bin level increased, but were still reduced (the power of detecting the change at the bin level was 23.7%.). These results confirm that effects introduced at more specific functional levels are likely to be diluted to the extent that they are hard to identify based on the quantitative information generated by traditional binning strategies. There is thus a significant gain of statistical power in performing the analysis at a more refined functional level, where the effect of dilution (i.e., the impact of the sequences that are not changing in abundance, or changing in another direction) will be reduced.

Changes in the abundance of biochemical functions and pathways between metagenomes reflects the microbial community response to a perturbation or change in environment. These changes are caused, explicitly or implicitly, by selection pressures acting on specific microbial phenotypes. The exact nature of these selection pressures is in most situations complex affecting both general and more specific biological functions in the microbial community. The stringency of the sub-bins derived by HirBin should therefore be based on underlying hypothesis and decided based on whether general or more specific effects are in focus. As a consequence, the stringency cutoff has been made adaptable and can be set by specifying the minimum amino acid similarity allowed for the functional domains defining each sub-bin. More stringent sub-bins (higher sequence similarity) are thus in general more homogenous, both sequence-wise and functionally. It is however important to note that the stringency of the sub-bins will affect the number of associated sequence reads, i.e., the coverage of the annotated regions. Since sub-bins are formed by splitting less specific bins and sub-bins, the number of sequence reads will decrease with increased stringency. Both analyzed datasets showed almost a ten-fold decrease in the average abundance of the sub-bins (75% sequence similarity) compared to the general bins (Additional file 7). The 112 bins that were not detected by HirBin at the 50% sub-bin level, but were significant at the bin level all had a relatively low average abundance, and are therefore harder to detect when splitting up the bins to sub-bins. The ability of detecting differential abundance is dependent on the total read count and an increased stringency will thus result in a reduction of the statistical power. Many modern metagenomes are however sequenced at high depths in order to ensure a satisfactory *de novo* assembly of the reference [29, 30] and it is very likely that this results in enough sequence reads for a suitable statistical power, even for the more stringent sub-bins. However, the reduction of power at more specific levels will not be as drastic for metagenomic datasets with a high number of biological samples. However, for less comprehensively characterized metagenomes with a few number of samples, the sub-bin stringency should be set with sequencing depth in mind. Furthermore, applying a high stringency cut-off may result in sub-bins formed around sample-specific gene variants. This phenomenon was seen in both human gut datasets analyzed in this study where the number of sub-bins classified as representative (present in at least 75% of the samples) decreased with increasing stringency (a drop from 15,740 to 10,798at a sequence similarity of 50 and 75% respectively). These results are in concordance with the previously reported high diversity of the human gut microbiome between individuals where the overlap at more specific taxonomic levels often is small [25, 26, 31]. Thus, metagenomes that are genetically diverse will, in general, exhibit a lower rate of stringent sub-bins present in the majority of the samples. The sequence similarity cutoff in the sub-binning step should thus be chosen in a way to assure that the clusters are large enough to be representative over the samples, but still specific enough to capture the effect at the right functional level.

Previous shotgun metagenomics studies, both from the human microbiome and environmental samples have shown that natural microbial communities are very complex and show a high diversity, both at the species and, consequently, the functional level [25, 32, 33]. In order to capture the change in abundance between different samples in the binning process it is therefore necessary to first capture the high diversity present in each functional domain. Profile databases such as PFAM and TIGRFAM have been specifically designed to be as broad as possible and thus to include a large number of taxonomically diverse gene variants [16, 17]. As a result, these domains are general classifications of functions. We show in this paper, that for many functional domains only subsets of the annotated sequences change in abundance, which means that the effect might be missed when comparing all the reads associated with that domain. The two-step procedure in HirBin makes it possible to detect those changes by first performing a broader annotation and then zooming in at more specific clusters of the functional domain (in the sub-binning step), giving a higher resolution in the binning process. It should, however, be emphasized that HirBin does not provide any refined functional information, since the sub-bins are annotated with the same function as their parent bin. HirBin will, however, identify functional differences that are only present at the higher resolution. For selected sub-bins it is possible to align the sequences to a reference database for further investigation of their function (can be done directly using the HirBin function blastSubbinSequences). For example, when the sub-bins for TIGR03537 in Fig. 3a are compared to GenBank (nr database) we found that the sequences in sub-bins 1 and 2 (at 50% sequence similarity) matched different variants of the aminotransferases (Additional file 8). All sequences in sub-bin 1 annotates as histidinol-phosphate transaminase (HisPAT, E.C. 2.6.1.9) involved in histidine biosynthesis [34] while the sequences in sub-bin 2 annotates as LL-diaminopimelate aminotransferase (LL-DAP-AT, E.C. 2.6.1.83), involved in lysine biosynthesis [35]. This shows an example where HirBin was able to separate two functionally different gene variants, and identify one as increasing and one as decreasing in abundance between the studied conditions. It should be noted that the HisP aminotransferases in sub-bin 1 were annotated mainly in bacteria from the *Bacteroidetes* phylum (in *Bacteroides* sp. and *Prevotella* sp.), while the LL-DAP aminotransferases in sub-bin 2 were mainly found in the *Firmicutes* phylum (in *Coprococcus* sp., *Clostridium* sp. and *Eubacterium* sp.). The observed changes in abundance for these could therefore be an indirect effect of changes in the taxonomic composition between t2d and control and does not necessarily reflect a direct impact on the aminotransferases. We argue, however, that it is important to be able to differentiate between these variants in the binning process, in order to identify differentially abundant functions, and facilitating the interpretation of the data.

The ability of detecting differentially abundant bins and sub-bins is dependent on the reference sequences. For many metagenomes, comprehensive catalogs of representative genomes and genes are lacking or completely missing, and contigs assembled de novo from the data, are therefore typically used as references [33]. The approach used by HirBin is general and can be applied to most types of references. HirBin can also be combined with different types of databases for annotation of the reference. For the datasets analyzed in this study, we used HMM-based annotation using the TIGRFAM database, which contains a comprehensive catalogue of bacterial functions. It is, however, possible to generate annotation based on other functional domains (e.g., PFAM and FOAM) or by BLAST-based sequence alignments against protein databases (e.g., KEGG Orthologies). Functional annotation of the reference sequences can be performed using the HirBin function functionalAnnotation, using a database of choice. The annotation can also be supplied by the user as a tab-separated annotation file with coordinates of any functional annotation. This makes it also possible to combine HirBin with many of the existing binning algorithms. Furthermore, the gene quantification in HirBin is done by matching reads against the reference sequence. HirBin can use a variety of mappers for this purpose, such as bowtie2 [36], or mappers using distributed computing for large-scale metagenomes, such as Tentacle [13]. HirBin is thus highly adaptable and should hence be applicable to a wide range of metagenomes and experimental designs.

HirBin uses sample-wide clustering of the functional domains to identify the sub-bins. An alternative approach would be to instead cluster the individual reads matching each functional domain. This clustering problem is however substantially harder due to the short length of the reads generated by the current sequencing technology (most modern metagenomes have a read length between 100 and 150 bases) and the often high error rate [37]. Furthermore, the number of reads are larger than the number of proteins, which also would increase the computational complexity. The clustering of functional domains in HirBin was done by UCLUST, which employs a centroid-based algorithm that identify clusters with sequences with a similarity above a specified cut-off [38]. UCLUST highly efficient and capable to calculate the sub-bins within an acceptable timeframe (<1 h), even for a large number of protein sequences (there were e.g., 23 million functional domains in the data from Qin et al. [25]). UCLUST have been shown to have an overall good and robust performance when clustering a large number of sequences [39]. It should however be pointed out that UCLUST does not perform hierarchical clustering and there is thus no guarantee that sub-bins at different sequence similarity cutoffs are formed as perfect subsets [40]. For the datasets in this study, this effect was present, but in general minor where 92.5% of the sub-bins were perfect subsets of a less stringent sub-bin. Replacing UCLUST with e.g., agglomerative hierarchical clustering based on complete linkage, would result in sub-bins that are more homogenous and strictly monotonously decreasing with increased stringency [41]. In order to compare UCLUST with hierarchical clustering we compared the number of sub-bins produced when clustering the sequences in 4 selected bins using both methods (Additional file 9). Hierarchical clustering resulted in a moderate increase in the number of sub-bins (on average 31%). However, the computational time was up to 2000 times longer using hierarchical clustering due to the high computational complexity (O(n^3) for agglomerative hierarchical clustering). Thus, UCLUST constitutes a good compromise between run-time and cluster quality and enables HirBin to be applied to very large reference databases.

## Conclusions

We present HirBin, a novel method for gene-centric analysis of metagenomics data. HirBin extends the standard supervised binning with an unsupervised clustering step, which enables quantification of metagenomes at a sub-bin level. This makes it possible to identify changes at a more specific functional level than what is possible by using traditional methods. HirBin is therefore useful for studying complex metagenomic datasets where it can facilitate the data interpretation and generate results that are more biologically relevant. HirBin is freely available at http://bioinformatics.math.chalmers.se/hirbin.

## Methods

HirBin is implemented as a Python package and is available at http://bioinformatics.math.chalmers.se/hirbin. HirBin makes use of the stand-alone tools Transeq v.6.3.1 (http://www.ebi.ac.uk/emboss/transeq/), HMMER v.3.1b1 [42] and UCLUST v 8.0 [38] for functional annotation and clustering. The quantification of the bins and sub-bins can either be done using bowtie2 [36] together with the bedtools function coverageBed [43], or using Tentacle [13]. The statistical analysis requires R to be installed (https://cran.r-project.org/). HirBin can be used for the entire analysis including supervised functional annotation (binning), unsupervised clustering of the bins (sub-binning), quantification and statistical analysis. Alternatively, the user can input their own annotation of the reference sequence in gff format, and their own quantification and use HirBin only for sub-binning and statistical analysis. Since HirBin also outputs an abundance matrix with the counts of all sub-bins it is also possible to use any method for statistical analysis, making the HirBin framework highly flexible and adaptable.

### Functional annotation (supervised)

For a given dataset HirBin inputs reference sequences in FASTA format (typically contigs from a metagenome assembly) and sequence reads in FASTQ format. The first step is the supervised functional annotation of the reference sequences using the HirBin sub-routine functionalAnnotation.py. The default annotation is based on TIGRFAMs, but it is possible to use other databases, e.g., PFAM [16] or COGs [19]. Using the default annotation procedure, the reference sequences were translated into all 6 possible reading frames and then annotated using the TIGRFAM database, release 13.0 [17] using HMMER v.3.1b1 [42], with e-value cutoff 1e-10 and output format -domtblout. The output file from HMMER was then parsed using the HirBin method extractSequences.py and a fasta file with protein sequences of the annotated domains was created to be used as input to the clustering step. The HirBin function extractSequences.py includes a parameter -maxAcceptableOverlap which specifies if multiple domains should be annotated at the same region of the reference sequence. If the parameter *p* is set to 1, all domains with an e-value lower than the threshold are reported and if it is lower than 1, only the best scoring annotation is reported if two annotations overlap each other more than *p* percent of the total length of the annotation. The coordinates of the annotated genes were converted from amino acid sequences to nucleotide reference coordinates, to facilitate in mapping the raw reads to the reference sequence for each annotated domain.

### Unsupervised clustering

The protein sequences belonging to each TIGRFAM domain is, for a given amino acid sequence similarity cutoff clustered by HirBin using either UCLUST (cluster_fast in USEARCH v8.0) or agglomerative hierarchical clustering (cluster_agg in USEARCH v8.0) [38]. UCLUST was used for all results presented in the paper. The cluster structure was saved into a file to be used later in the analysis.

### Quantification and statistical analysis of sub-bins

The abundance of each domain was calculated by mapping the Illumina reads for each sample to the annotated regions of the reference sequence. Bad quality sequences were filtered out from the fastq files prior to mapping, by removing reads where at least 50% of the bases had a quality score less than 10. The quantification can be performed by combining alignment using bowtie2 [36] with the bedtools function coverageBed [43], or using Tentacle v.0.1 [13] and pBlat v.35 (http://icebert.github.io/pblat/) [44] using command line arguments “-threads 16 -minIdentity 90 -out = blast8”. The mapping results were incorporated into the cluster structure, using the HirBin subroutine calculateSubBins.py forming sub-bins. The output from this step was an abundance matrix with the number of sequences that match each sub-bin, in each sample. In order to assure that all sub-bins are representative across many samples, HirBin kept only the sub-bins that are represented in at least 75% of all individuals, and the small sub-bins were filtered out (this criteria can be changed if needed). Statistical analysis was performed in R using an over-dispersed Poisson generalized linear model (GLM) with a log-likelihood link, similar to the model used in [45]. This model has been shown to perform well in the analysis of gene abundances in metagenomes in comparison with other statistical models [8]. The *p*-values were corrected for multiple testing using Benjamini and Hochberg’s false discovery rate (FDR) [46].

### Analysis of metagenomics data for type 2 diabetes vs. control

Metagenomics sequences of the gut microbiota of 15 diabetic male individuals and 15 healthy male individuals were randomly chosen from the Qin et al. (2012) [24] study. The sample names of the 30 selected samples are available in Additional file 10. Raw Illumina data was downloaded from the NCBI short read archive (SRA) and assembled sequences for each sample was downloaded from ftp://climb.genomics.cn. The data was analyzed with HirBin as described in the previous sections, including annotation of the assembled sequence contigs to the TIGRFAM database using e-value cutoff 1e-10 and-maxAcceptableOverlap = 1 (supervised annotation), clustering of each TIGRFAM domain (unsupervised), mapping and calculation of sub-bins. The total number of sequences to be clustered was 3,425,805. The unsupervised clustering step was performed two times using 50 and 75% sequence identity cutoffs, representing a less strict and a stricter clustering. (. The clustering took less than 20 min to perform using 12 CPU cores. In order to find which sub-bins that overlapped at 50 and 75% sequence identity cutoffs the UCLUST output files were investigated to find the sub-bins with overlapping contig IDs. To identify significantly over-represented GO-terms based on the TIGRFAM significance, integrative analysis was performed for each bin/sub-bin level using Gene set enrichment analysis (GSEA) [27] as implemented in the PIANO R-package [47]. As input to the GSEA method a ranked list of bins/sub-bins with positive log fold change and a ranked list of bins/sub-bins with negative log fold change was created, based on the FDR. The links between TIGRFAMs and GO-terms were obtained from TIGRFAM v. 13.0. The GSEA parameter of the running sum was set to 1. The GSEA *p*-value was calculated by performing 1000 permutations.

### Evaluation of statistical power on resampled data

Metagenomic data from the Qin et al. (2010) [25] study was downloaded from http://gutmeta.genomics.org.cn/ including both raw sequence reads and sample-specific metagenomics assemblies for the gut metagenomes of 124 individuals. The data was analyzed as before with HirBin forming sub-bins from each TIGRFAM domain. The resampling was done at a given sequence identity clustering level (between 50 to 80%) by first selecting 30 random samples and divide them into two groups. An effect was introduced to 10% of the sub-bins by down-sampling of the reads in one of the groups, in the same way as in Jonsson et al. (2016) [8]. The observed read count of each contig in the sub-bin, *Y*_*ij*_ (sub-bin *i* in sample *j*) was replaced with a downsampled value *Y*_ij_^*^ drawn randomly from a binomial distribution with parameters *Y*_*ij*_ and 1/7. The down-sampling resulted in an average fold change of 7 between groups. In this way the variance structure of the metagenome data is maintained. The whole resampling procedure was repeated 100 times creating 100 separate data sets. Since the down-sampling was done at the contig level, the effect could be tracked at the other clustering levels by a Python script, using the clustering structure. In each round of iteration, a sub-bin at any clustering level was considered to be affected if at least one of the contigs was affected (i.e., if the sub-bin overlapped a down-sampled sub-bin at the 80% sequence identity clustering level). The statistical power at each clustering level was calculated as the ability to detect the effects introduced at the sub-bin level.

## Additional files

Additional file 1: Results from the statistical analysis of bins and sub-bins for the t2d (Qin [24]) dataset. (XLSX 2318 kb)

Additional file 2: Figure showing the mean abundances of the bins that are significant at the bin level. (PDF 5 kb)

Additional file 3: Results from the gene set enrichment analysis of GO-terms for bins and sub-bins. (XLSX 584 kb)

Additional file 4: Figure showing the sub-bin profile for all sub-bins in the two example bins in Fig. 3. (PDF 4 kb)

Additional file 5: Table showing the number of bins and sub-bins at different sequence identity cutoffs for the Qin [25] dataset. (XLSX 8 kb)

Additional file 6: Figure showing Fold changes at the bin level when the resampling is performed at different sub-bin levels. (PDF 24 kb)

Additional file 7: Histogram showing mean abundances of the bins and sub-bins for the Qin [24] dataset. (PDF 5 kb)

Additional file 8: Table showing annotations of the sequences of TIGR03537, sub-bin 1 and 2 at 60% sequence similarity. (XLSX 21 kb)

Additional file 9: Comparison between UCLUST and hierarchical clustering. Table showing the number of clusters and computational times using both methods for 4 selected bins in the Qin [24] dataset. (XLSX 8 kb)

Additional file 10: Table showing selected samples from the Qin [25] study for the resampling analysis. (XLSX 8 kb)

## Abbreviations

FDR: False Discovery Rate
GO: Gene Ontology
GSEA: Gene set enrichment analysis
HirBin: High-Resolution Binning
HMM: Hidden Markov Model
KO: KEGG Ortology
t2d: Type 2 diabetes
