# Supplementary figures and images for: HirBin: high-resolution identification of differentially abundant functions in metagenomes
Source: BMC Genomics. 2017 Apr 21;18:316. doi: 10.1186/s12864-017-3686-6 (PMC5399828; doi:10.1186/s12864-017-3686-6)

## Mean abundance of bins

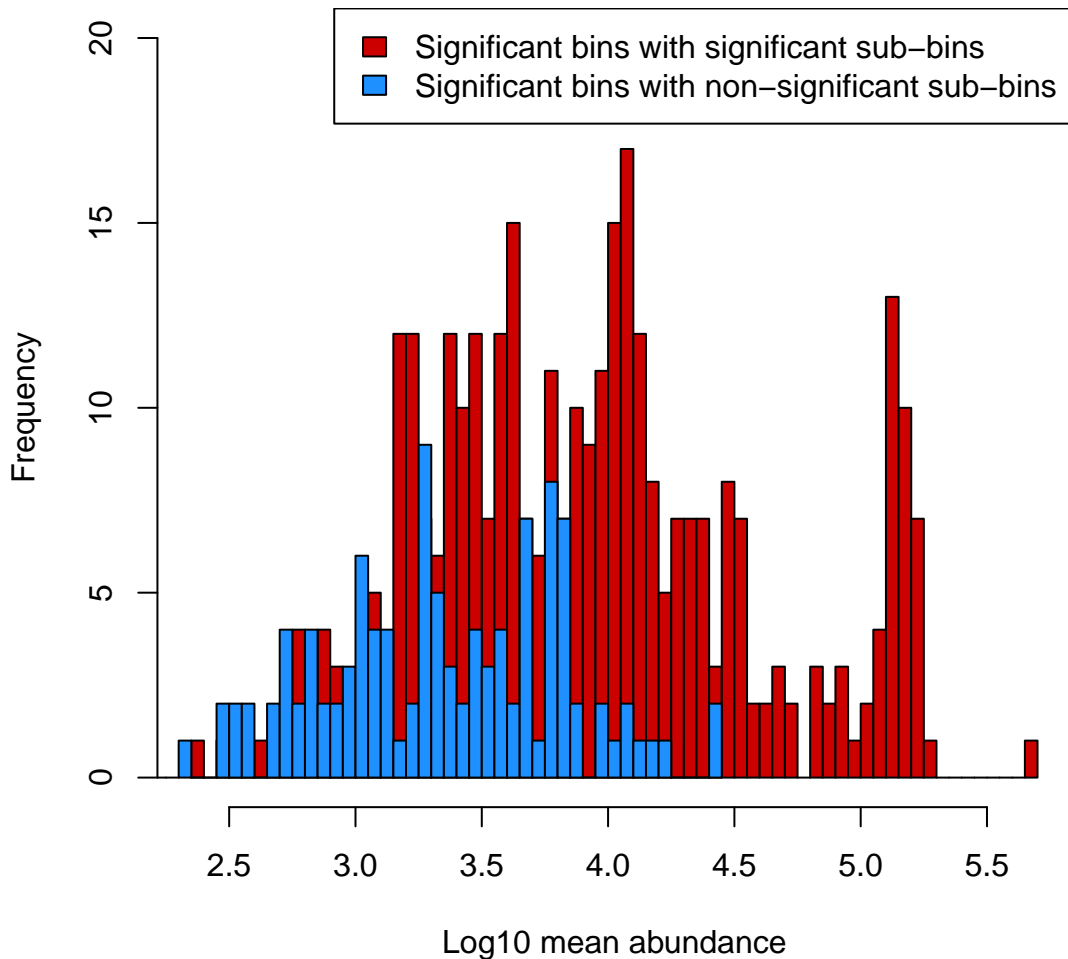

Supplement: Supplementary file 2 — Figure showing the mean abundances of the bins that are significant at the bin level. (PDF 5 kb) [file 12864_2017_3686_MOESM2_ESM.pdf]

**TIGR03537**

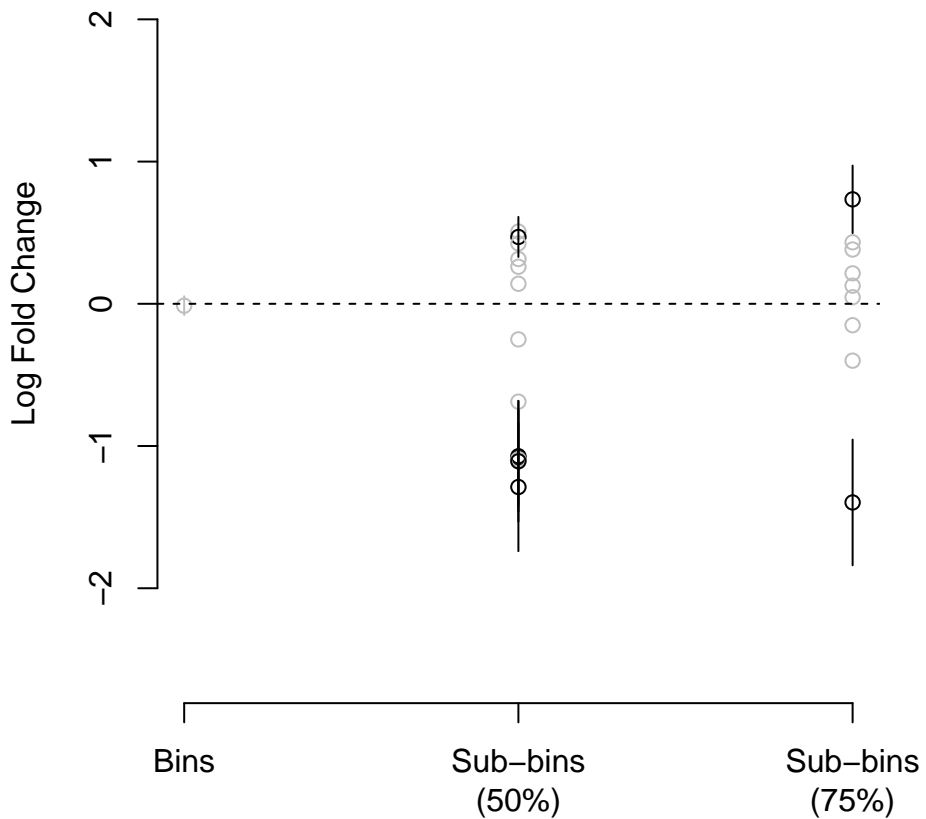

**TIGR01243**

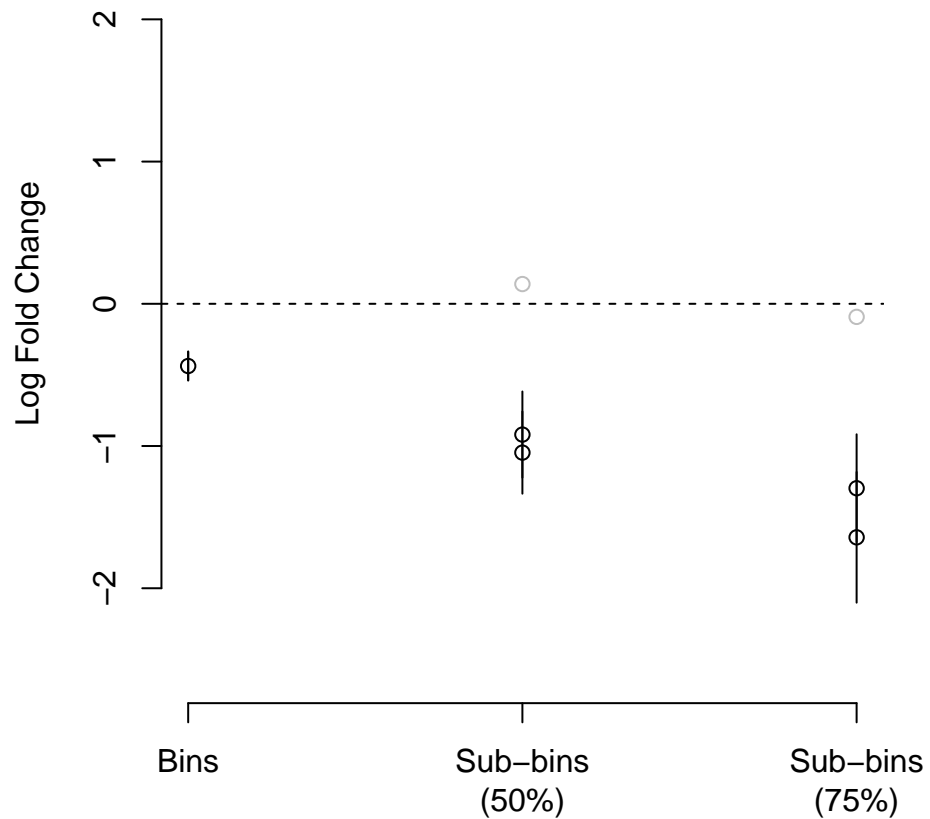

Supplement: Supplementary file 4 — Figure showing the sub-bin profile for all sub-bins in the two example bins in Fig. 3. (PDF 4 kb) [file 12864_2017_3686_MOESM4_ESM.pdf]

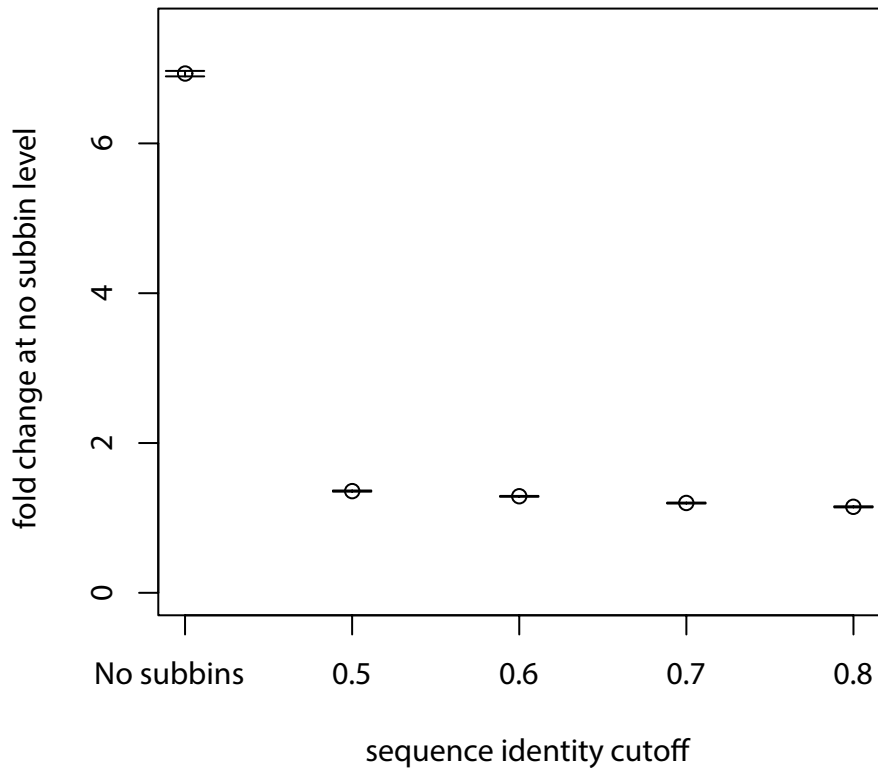

Supplement: Supplementary file 6 — Figure showing Fold changes at the bin level when the resampling is performed at different sub-bin levels. (PDF 24 kb) [file 12864_2017_3686_MOESM6_ESM.pdf]

## Mean abundance of bins

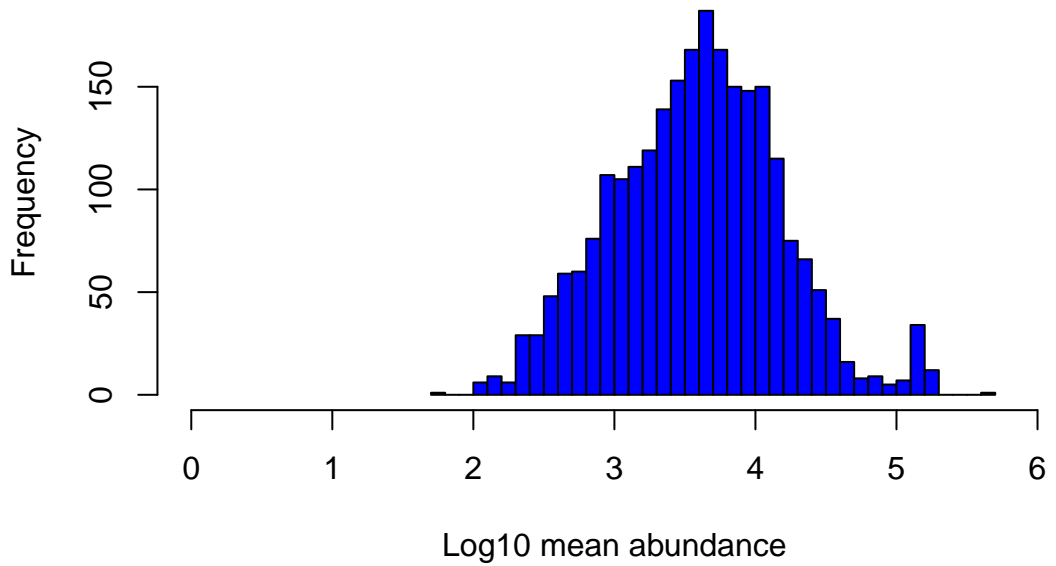

## Mean abundance of sub-bins at 75% similarity

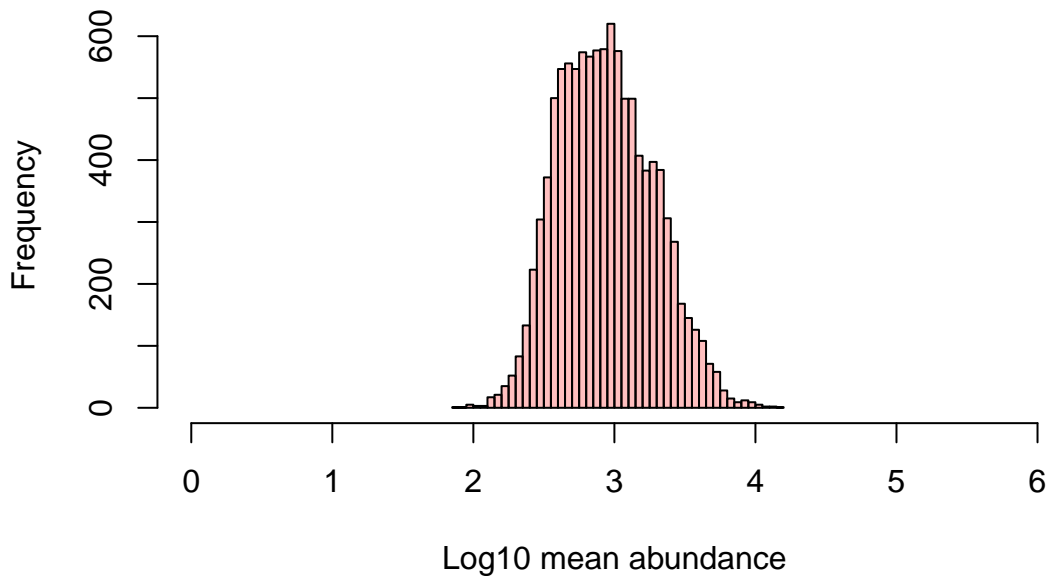

Supplement: Supplementary file 7 — Histogram showing mean abundances of the bins and sub-bins for the Qin [24] dataset. (PDF 5 kb) [file 12864_2017_3686_MOESM7_ESM.pdf]
